# Validation of *in vivo* 2D displacements from spiral cine DENSE at 3T

**DOI:** 10.1186/s12968-015-0119-z

**Published:** 2015-01-30

**Authors:** Gregory J Wehner, Jonathan D Suever, Christopher M Haggerty, Linyuan Jing, David K Powell, Sean M Hamlet, Jonathan D Grabau, Walter Dimitri Mojsejenko, Xiaodong Zhong, Frederick H Epstein, Brandon K Fornwalt

**Affiliations:** Department of Biomedical Engineering, University of Kentucky, 741 S Limestone, BBSRB B353, Lexington, KY 40509 USA; Department of Pediatrics, University of Kentucky, Lexington, USA; Department of Electrical Engineering, University of Kentucky, Lexington, USA; MR R&D Collaborations, Siemens Healthcare, Atlanta, GA USA; Department of Biomedical Engineering, University of Virginia, Charlottesville, VA USA; Departments of Physiology and Medicine, University of Kentucky, Lexington, USA

**Keywords:** DENSE, Displacement, Cardiac mechanics, Spiral, Strain, Torsion, 3T, Cardiovascular magnetic resonance

## Abstract

**Background:**

Displacement Encoding with Stimulated Echoes (DENSE) encodes displacement into the phase of the magnetic resonance signal. Due to the stimulated echo, the signal is inherently low and fades through the cardiac cycle. To compensate, a spiral acquisition has been used at 1.5T. This spiral sequence has not been validated at 3T, where the increased signal would be valuable, but field inhomogeneities may result in measurement errors. We hypothesized that spiral cine DENSE is valid at 3T and tested this hypothesis by measuring displacement errors at both 1.5T and 3T *in vivo*.

**Methods:**

Two-dimensional spiral cine DENSE and tagged imaging of the left ventricle were performed on ten healthy subjects at 3T and six healthy subjects at 1.5T. Intersection points were identified on tagged images near end-systole. Displacements from the DENSE images were used to project those points back to their origins. The deviation from a perfect grid was used as a measure of accuracy and quantified as root-mean-squared error. This measure was compared between 3T and 1.5T with the Wilcoxon rank sum test. Inter-observer variability of strains and torsion quantified by DENSE and agreement between DENSE and harmonic phase (HARP) were assessed by Bland-Altman analyses. The signal to noise ratio (SNR) at each cardiac phase was compared between 3T and 1.5T with the Wilcoxon rank sum test.

**Results:**

The displacement accuracy of spiral cine DENSE was not different between 3T and 1.5T (1.2 ± 0.3 mm and 1.2 ± 0.4 mm, respectively). Both values were lower than the DENSE pixel spacing of 2.8 mm. There were no substantial differences in inter-observer variability of DENSE or agreement of DENSE and HARP between 3T and 1.5T. Relative to 1.5T, the SNR at 3T was greater by a factor of 1.4 ± 0.3.

**Conclusions:**

The spiral cine DENSE acquisition that has been used at 1.5T to measure cardiac displacements can be applied at 3T with equivalent accuracy. The inter-observer variability and agreement of DENSE-derived peak strains and torsion with HARP is also comparable at both field strengths. Future studies with spiral cine DENSE may take advantage of the additional SNR at 3T.

## Background

**Figure 1:**
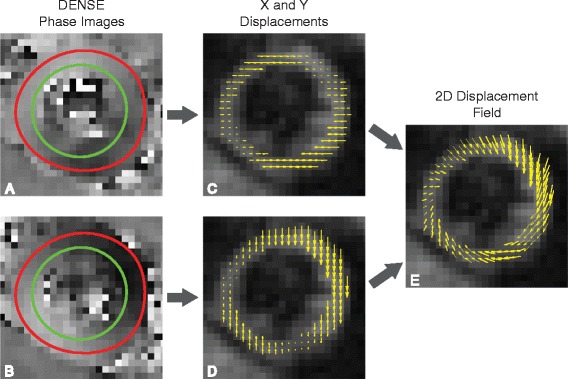
**Two-dimensional displacements measured by DENSE.** The first column **(A,B)** contains X and Y phase images obtained during systole for a healthy volunteer. The red and green contours define the epicardial and endocardial borders of the left ventricle, respectively. Each pixel within each phase image represents the Eulerian displacement in a single direction **(C,D)**. The displacements are overlaid on the magnitude image from DENSE. The third column **(E)** contains the 2D Eulerian displacement field that results from vector addition.

Displacement Encoding with Stimulated Echoes (DENSE) is a cardiovascular magnetic resonance (CMR) technique that encodes tissue displacement into the phase of the magnetic resonance signal [1]. This provides pixel-level resolution of Eulerian displacements throughout the imaged slice (Figure 1). Due to the stimulated echo acquisition, cine DENSE has inherently low signal that fades through the cardiac cycle as a result of T1 relaxation [1,2]. To counter these limitations, many studies with DENSE at 1.5T have employed a spiral acquisition, which efficiently acquires k-space and increases SNR compared to typical Cartesian strategies [3-6]. This acquisition has not been validated at 3T, where the benefits of further increased SNR and longer T1 relaxation times may be offset by field inhomogeneities and off-resonance artifacts that are likely more pronounced at the higher field strength.

Validation of DENSE has been performed at 1.5T in several ways: by comparing measured displacements to known displacements in a rigid rotating phantom [2,7], by comparing measured radial and shear strains to known strains in a non-physiologic deforming phantom [3], and by comparing left ventricular (LV) strains in volunteers quantified from DENSE to those quantified from myocardial tagging [3,8,9]. These validations and subsequent applications have led to the acceptance of spiral cine DENSE at 1.5T. This study extends those validations by using myocardial tagging to validate physiologic LV displacements and strains from spiral cine DENSE in human volunteers at 3T. By using human volunteers, rather than cylindrical phantoms, the realistic field inhomogeneities and off-resonance effects that are present at 3T can be investigated.

In addition to the accuracy of the DENSE displacements and strains, the SNR throughout the cardiac cycle is of interest. Studies with 2D spiral cine DENSE at 1.5T have used a constant flip angle strategy of 20° [3,10,11] or 15° [12]. However, 3D volumetric spiral cine DENSE has been performed at 1.5T with a ramped flip angle strategy [5], which tends to equalize the SNR across all cardiac phases by using lower flip angles early in the cardiac cycle [13]. A previous study has compared some flip angle strategies at 3T and 1.5T for cine DENSE with segmented echo planar imaging (EPI) [14], but a similar study has not been done for spiral cine DENSE.

We therefore aimed to test the following hypotheses: (1) DENSE at 3T has sub-pixel displacement accuracy in measuring physiologic motion and is not significantly different from the error at 1.5T, (2) the inter-observer variability of strains derived from DENSE at 3T is similar to that of DENSE at 1.5T, (3) the cardiac strains and torsion from DENSE at 3T agree with results from analyzing tagged images from the same locations using harmonic phase (HARP), and (4) the SNR of DENSE at 3T is higher than that at 1.5T and may be best leveraged with different flip angle strategies from those commonly used at 1.5T.

## Methods

### 3T imaging

**Figure 2:**
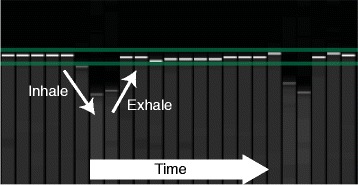
**Respiratory navigator feedback.** The image of the respiratory navigator that was projected to the volunteers in real-time during the DENSE scans. The horizontal green lines define the ±3 mm acceptance window while the bold white and gray tick marks define the diaphragm location. A breath (inhale and exhale) is labeled.

This protocol was approved by the Institutional Review Board of the University of Kentucky. Ten subjects (40% female, age 29 ± 4) with no history of cardiovascular disease were consented. Acquisitions at 3T were performed on a Siemens (Erlangen, Germany) Tim Trio with a 6-element chest and 24-element spine coil. After the standard localizers, a cardiac-gated field map was acquired during a breath-hold for second-order shimming. Three short-axis (base, mid, apex) and one long-axis (four-chamber) 2D spiral cine DENSE slices were acquired with the following parameters: 6 spiral interleaves (2 interleaves acquired per temporal frame), 360 × 360 mm^2^ field of view, 128 × 128 image matrix (2.8 × 2.8 mm^2^ pixel size), 8 mm slice thickness, 1.08 ms echo time, 17 ms repetition time (34 ms temporal resolution), 4 ms duration of spiral readout, 20° constant flip angle, 0.10 cycles/mm encoding frequency, simple encoding [10], 0.08 cycles/mm through-plane de-phasing frequency [11], and CSPAMM echo suppression [8]. The spiral acquisition yielded k-space data with a matrix size of 102, which was then zero-padded to 128. The two encoded dimensions were in-plane. The through-plane component was not acquired. Using the R-R interval from the real-time electrocardiogram (ECG), the number of cardiac phases was adjusted to have 100 ms to 150 ms of dead time, which refers to the period between the last acquired cardiac phase and the next QRS complex on ECG. Reconstruction was performed online with gridding and linear inhomogeneity compensation [5,15]. No additional off-resonance corrections were performed in the reconstruction. To remove the possible effects of variable breath-hold position, all DENSE scans were performed with a respiratory navigator (acceptance window ± 3 mm). In an effort to improve navigator efficiency, a real-time video of the navigator was projected to the subjects, which allowed them to adjust their diaphragm position and to maximize the time spent acquiring data when the diaphragm was located within the acceptance window (Figure 2).

During the same imaging session, tagged images were acquired at the same slice locations as DENSE with the following parameters: grid tagging 45° to readout direction, 8 mm tag spacing, 340 × 340 mm^2^ field of view, 256 × 256 acquisition and image matrices (1.3 × 1.3 mm^2^ pixel size), 8 mm slice thickness, 2.72 ms echo time, 5.72 ms repetition time, 15 segments, 10° constant flip angle, and 20 cardiac phases. The tagged images were acquired with pre-navigated breath-holds. The subjects used the same navigator as above (Figure 2) to place their diaphragm within the acceptance window. The operator watched the diaphragm position in real-time along with the subject and triggered the tagged acquisition once it was inside the acceptance window. The subjects then held their breath for the duration of the tagged acquisition.

### 1.5T imaging

Six of the ten subjects returned after 185 ± 76 days to perform similar scans at 1.5T on a Siemens Aera with an 18-element chest and 12-element spine coil. The DENSE acquisition parameters were the same as for the 3T case. As before, a respiratory navigator with a ±3 mm acceptance window was used. However, no visual feedback was available to be projected to the subjects. Myocardial tagging acquisition parameters were the same as for the 3T case with the following exceptions: 360 × 293 mm^2^ field of view, 256 × 141 acquisition matrix interpolated to 256 × 208 image matrix (1.4 × 1.4 mm^2^ pixel size), 3.89 ms echo time, 4.5 ms repetition time, 9 segments, 14° constant flip angle, and 15–21 cardiac phases (dependent on subject’s heart rate).

### Overview of displacement validation method

**Figure 3:**
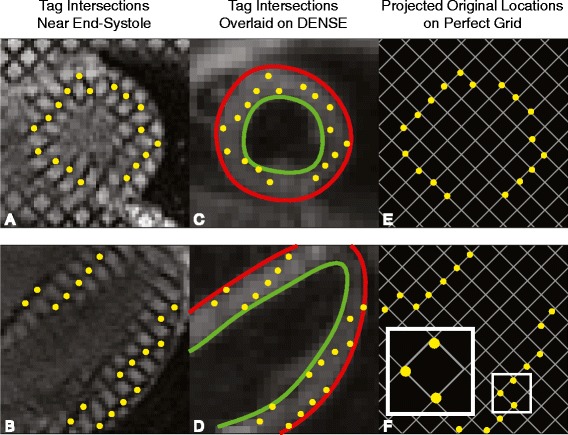
**DENSE displacements project tag intersection points back into a perfect grid.** By end-systole, a perfect grid of tag intersection points has deformed into a warped grid as seen in the first column for both a short-axis and four-chamber slice **(A,B)**. The deformed intersection points can be overlaid on DENSE images taken at the same point in the cardiac cycle **(C,D)**. The Eulerian displacements from the DENSE images can be used to project the tag intersection points back into a nearly perfect grid **(E,F)**. Deviation from a perfect grid of 8x8 mm^2^ is a measure of the error in DENSE displacements. The small box in **F** is enlarged to show an example of deviation from the nearest grid intersections.

The pixels within the phase images of DENSE represent the Eulerian displacements of the underlying tissue [1]. These displacements can be used to project the instantaneous locations of the tissue back to their original position during the encoding step, which is immediately after detection of the QRS complex on ECG. The encoding step of DENSE and the placement of a perfect grid of taglines in tagged imaging occur at the same point in the cardiac cycle. In myocardial tagging, this grid then deforms with the contracting tissue and the tag intersection points no longer form a perfect grid (Figures 3A, 3B). If a set of DENSE phase images (with in-plane displacements, denoted as X and Y) were acquired in the same spatial and temporal location as the image of the deformed grid, then the Eulerian displacements represented by the DENSE images could be used to project the tag intersection points back into the original, perfect grid. The deviation of these projections from a perfect grid is a measure of the accuracy in the DENSE displacement data. Figure 3 presents an example workflow for both short- and long-axis slices.

### Displacement validation

Displacement analysis was performed offline using custom software written in Matlab (The Mathworks Inc, Natick, MA). Four slices (base, mid, apex, and four-chamber) of myocardial tagging were acquired on each of the ten subjects at 3T and each of the six subjects at 1.5T. For each slice, a single observer manually identified the three cardiac phases nearest to end systole and then manually marked tag intersection points in the left ventricle on those cardiac phases. The observer was instructed to only mark definitive intersection points and to ignore intersection points that were unclear. The observer also supplied epicardial contours for each phase to be used for registering the tagged images to the DENSE images to account for any in-plane patient motion. Endocardial contours were not used due to difficulties in discriminating between papillary muscles, trabeculations, and the LV myocardium.

DENSE image processing included manual segmentation of the left ventricular myocardium and semi-automated phase unwrapping [5,7,16]. A single observer manually segmented the myocardium by providing endocardial and epicardial contours. Seed points (points that have not experienced phase wrapping) were supplied by the observer at the beginning of the semi-automated phase unwrapping. Using the displacement encoding frequency (0.10 cycles/mm), the unwrapped phase image data were converted to Eulerian displacement maps in millimeters.

Due to the differences in repetition times, DENSE and tagged images were not acquired at the same time points during the cardiac cycle. Therefore, for each slice, the DENSE cardiac phase that was closest in time to one of the marked tagged images was used for further analysis. The tagged image that approximated that DENSE cardiac phase in time was also used for further analysis.

To account for any in-plane patient motion between the tagged and DENSE images, the centroids of the epicardial contours were aligned. The marked tag intersections were then used as points to sample the X and Y Eulerian displacement maps. Linear interpolation was used to determine the X and Y Eulerian displacements for each tag intersection point. Those displacements were then used to project the intersection points back to their initial, pre-deformed location. Ideally, these projected points would have formed a perfect grid, which is the initial configuration of the tag points when they were applied. Importantly, no other smoothing or processing of the DENSE displacements was performed. Aside from the phase unwrapping and linear interpolation, this method provided a true investigation of the raw displacements from the DENSE imaging.

To measure the deviation from a perfect grid, an 8 × 8 mm^2^ grid at a 45° angle was constructed with reference points located at the grid intersections. These grid parameters are identical to the parameters from the tagged acquisition. An iterative closest point algorithm [17] that did not permit rotation was used to fit this perfect grid to the projected tag intersection points and to calculate the root-mean-squared error (RMSE) between the projected tag intersection points and the nearest perfect grid reference points. The iterative closest point algorithm was used because the exact location of the 45° grid was unknown as no tagged images are acquired at the same instant as the encoding.

For each slice location (four-chamber, base, mid, apex), the distribution of RMSE across the subjects at 3T was compared to the distribution at 1.5T with the Wilcoxon rank sum test.

### Strain and torsion analyses

Strains from all DENSE slices were calculated by further processing in Matlab of the displacement-encoded phase images. Following automated phase unwrapping, spatial smoothing and temporal fitting of displacements were performed as described previously [5,7,16]. This processing provided smooth trajectories for all tissue points beginning at end-diastole (the time of DENSE encoding) and continuing through much of diastole. The trajectories were not extrapolated into the dead time (the last 100–150 ms of diastole that were not imaged).

Strains were then quantified with the 2-dimensional Lagrangian finite strain tensor. For each short-axis slice, peak radial and circumferential strains were calculated by averaging the strains from all segments within the slice and selecting the peak from the average strain curve. Radial strain was defined as positive for thickening while circumferential strain was negative for shortening. Peak longitudinal strain was calculated in a similar manner from the four-chamber DENSE slice. The most apical segment was excluded from the average before selecting the peak as previously reported [18]. Longitudinal strain was defined as negative for shortening.

In addition to strain, each short-axis DENSE slice also provided a measure of twist. Torsion was then calculated as the gradient of twist down the long axis of the LV by finding the slope of the linear regression line between twist and longitudinal position. The peak torsion was then selected from the torsion curve and reported in units of degrees/mm. Twist was defined as positive for counterclockwise rotation when viewing a short-axis slice from the apex towards the base. Torsion was positive when the apex was twisting more positively than the base.

Strains and twists from tagged slices were calculated with HARP (Diagnosoft, Durham, NC). For comparison with DENSE, Lagrangian strains were exported from the software for each segment around the myocardium. Twists were exported from HARP for each of the short axis slices and torsion was calculated in the same manner as above. As with DENSE, the most apical segment of the four-chamber slice was excluded from the longitudinal strain average. Other segments were also excluded on a case-by-case basis due to poor tracking. Poor tracking was assessed visually by the observer who was blinded to the results of the DENSE strain analyses.

Two observers independently analyzed each of the DENSE slices in order to compare inter-observer variability in strains and torsions at each field strength with Bland-Altman analysis [19]. A single observer analyzed the tagged slices with HARP for comparison of strains and torsion between DENSE and myocardial tagging at each field strength with Bland-Altman analysis and modified coefficient of variation (CoV) [18]. The equation for CoV for a variable, *X*, measured on *N* subjects by two observers is below.

$$ CoV=\frac{{\displaystyle {\sum}_{i=1}^N}\left[ St.Dev.{\left({X}_{Obs.1}\kern0.5em {X}_{Obs.2}\right)}_i\right]/N}{\left|{\displaystyle {\sum}_{i=1}^N}\left[{\left(\left({X}_{Obs.1}+{X}_{Obs,2}\right)/2\right)}_i\right]/N\right|} $$

### DENSE signal to noise ratio

To compare the SNR of DENSE between 3T and 1.5T, the SNR was calculated for each cardiac phase within the mid-ventricular slice of each subject. SNR was calculated from the magnitude images by averaging the signal within the myocardium and finding the standard deviation (noise) of signal within a region outside the body (air) with the care to avoid image artifacts. Corrections were applied for the Rician distribution of the magnetic resonance signal [20]. The true standard deviation of the signal, *σ*, was calculated from the measured standard deviation, *σ*_*M*_, by

$$ \sigma =\sqrt{\frac{2}{4-\pi }}*{\sigma}_M\approx 1.526*{\sigma}_M $$

The true myocardial signal, *S*, was calculated from the measured myocardial signal, *M*, by

$$ S=\sqrt{M^2-{\sigma}^2} $$

The DENSE sequences at 3T and 1.5T varied slightly in the way the magnitude images were reconstructed, likely due to the different versions of DENSE required for the different software installed on the 3T Tim Trio and the 1.5T Aera (Syngo MR B17 and Syngo MR D13, respectively). At 3T, the magnitude images within a cine series were not normalized independently. Thus, the noise from each cardiac phase was averaged together to get a single noise value for the entire series. The myocardial signal from each cardiac phase was then divided by this noise value to obtain SNR through the cine series. At 1.5T, the reconstruction normalized each image independently from others in the cine series, presumably to aid readers in viewing image contrast. Due to this independent scaling, the noise could not be considered constant across all cardiac phases. The SNR of each cardiac phase was calculated from its own noise level without averaging the noise of all phases together. The SNR at each cardiac phase was compared between 3T and 1.5T with the Wilcoxon rank sum test.

### DENSE flip angle analysis

**Figure 4:**
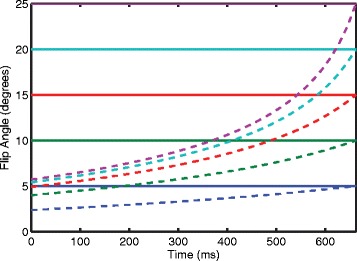
**Illustration of flip angle strategies throughout the cardiac cycle.** The bold, horizontal lines indicate the constant flip angle strategies where the same flip angle is applied throughout the cardiac cycle. The ramped flip angle strategies are indicated by the dashed lines and were calculated as described by Stuber *et al.* [13]. Notice that the ramped flip angle strategies start at low values and increase throughout the cardiac cycle. They are defined by their last flip angle (e.g. the red dashed line is the ramped flip angle strategy that ends at 15°). The ramped flip angle strategies in this illustration were calculated by using 17 ms repetition time, 1000 ms T1 relaxation constant, and 20 cardiac phases (40 repetition times). Typical values of myocardial T1 for healthy volunteers at 3T and 1.5T have been found [21] and were used in this illustration.

Two of the subjects underwent further DENSE imaging at 3T to assess the SNR of different flip angle strategies. Both constant and ramped flip angle strategies of 5, 10, 15, 20, and 25° were investigated [13] (Figure 4). The ramped flip angle strategies were designed to maintain equal SNR throughout the cardiac cycle [13]. For a given last flip angle, *α*_*N*_, the preceding flip angles can be calculated iteratively as below:

$$ {\alpha}_{n-1}={ \tan}^{-1}\left( \exp \left(\frac{-TR}{T1}\right)* \sin \left({\alpha}_n\right)\right) $$

TR is the repetition time while T1 is the relaxation constant. A single mid-ventricular short-axis slice was imaged in each case. All other DENSE acquisition parameters remained the same. The SNR of each strategy was calculated in the same manner as above for the 3T case. For each subject, the SNRs were qualitatively compared to investigate whether a strategy other than a constant 20° flip angle would be preferable at 3T.

## Results

### Displacement validation

At 3T, the average temporal difference between the DENSE and tagged images was 3.3 (range: 0.1 – 13.0) ms. At 1.5T, the average difference was 2.8 (range: 0.8 – 7.7) ms. Both times represent good temporal agreement between the analyzed DENSE and tagged images.

**Figure 5:**
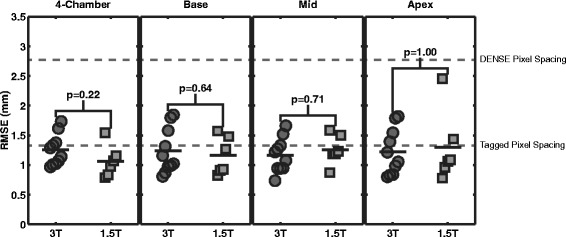
**RMSE for each slice orientation at 3T and 1.5T.** The error in DENSE displacements as measured by RMSE is shown for each type of slice. The top gray line indicates the DENSE pixel spacing of 2.8 mm. The bottom gray line was placed at 1.35 mm, which is the average of the tag pixel spacing at 3T and 1.5T (1.3 mm and 1.4 mm, respectively). The mean RMSEs were below the DENSE pixel spacing and were below or on the order of the tagged pixel spacing. No significant difference in RMSE were seen between 3T and 1.5T by the Wilcoxon rank sum test for any slice orientation.

The error in the DENSE displacements, as measured by the RMSE between the projected tag intersection points and a perfect grid, are presented in Figure 5. For each slice orientation, no significant differences was seen between the RMSE at 3T and the RMSE at 1.5T. Considering all slices together, the average RMSEs for 3T and 1.5T were 1.2 ± 0.3 mm and 1.2 ± 0.4 mm, respectively. All RMSEs were below the DENSE pixel spacing (2.8 mm) and below or on the order of the tagged pixel spacing (1.3 and 1.4 mm).

### Strain and torsion analyses

**Figure 6:**
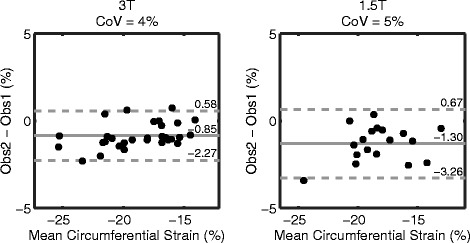
**Spiral cine DENSE has good inter-observer variability in circumferential strain at both 3T and 1.5T.** The Bland-Altman plots contain the inter-observer peak circumferential strain from the base, mid, and apex slices of DENSE for each field strength. The solid lines indicate the biases while the dashed lines are the 95% limits of agreement.

As a second comparison of spiral cine DENSE between 3T and 1.5T, the inter-observer variability in the peak strains and torsions produced by analyzing the DENSE slices was assessed with Bland-Altman analyses and CoV. Figure 6 contains the Bland-Altman figures for circumferential strain from the three short-axis slices. Both field strengths demonstrated good reproducibility between observers.

Table 1 contains inter-observer statistics for the remaining strains and torsion. At both field strengths, longitudinal strain and torsion demonstrated low CoVs. Radial strain, however, had higher biases, 95% limits of agreement, and CoVs compared to the other measures.

**Table 1:** **Inter-observer variability in strains and torsion quantified with spiral cine DENSE were similar at 3T and 1.5T**

	**3T**			**1.5T**		
	**Bias**	**95% Limits**	**CoV (%)**	**Bias**	**95% Limits**	**CoV (%)**
**Circumferential strain (%)**	−0.8	±1.4	3.6	−1.3	±2.0	5.2
**Radial strain (%)**	4.1	±14.7	10.5	−4.5	±13.3	10.4
**Longitudinal strain (%)**	0.4	±1.9	3.9	−0.8	±1.5	5.3
**Torsion (deg/mm)**	0.01	±0.02	2.9	0.02	±0.02	3.5

**Figure 7:**
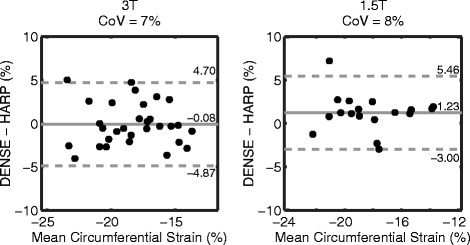
**Bland-Altman plots for circumferential strain between DENSE and HARP.** The solid lines indicate the biases while the dashed lines are the 95% limits of agreement. The 95% limits and CoVs between DENSE and HARP were larger than DENSE inter-observer variability.

Figure 7 contains the Bland-Altman analyses for circumferential strain between DENSE and tagged analyses with HARP. The 95% limits and CoVs were higher between DENSE and HARP than the same measurements of DENSE inter-observer variability.

Table 2 contains the Bland-Altman analyses comparing DENSE to HARP for the remaining strains and torsion. The two field strengths demonstrated comparable agreement between DENSE and HARP. For radial strain and torsion, the 95% limits of agreement and CoVs were high at both field strengths. Circumferential and longitudinal strains showed better agreement.

**Table 2:** **Variability in strains and torsion between DENSE and HARP at 3T and 1.5T (Bias: DENSE - HARP)**

	**3T**			**1.5T**		
	**Bias**	**95% Limits**	**CoV (%)**	**Bias**	**95% Limits**	**CoV (%)**
**Circumferential strain (%)**	−0.8	±4.8	7.5	1.2	±4.2	7.6
**Radial strain (%)**	−5.3	±40.0	28.5	3.4	±52.2	36.0
**Longitudinal strain (%)**	−1.8	±3.2	9.8	2.0	±5.6	13.0
**Torsion (deg/mm)**	0.13	±0.09	30.4	0.11	±0.12	31.6

### DENSE signal to noise ratio and flip angle analyses

**Figure 8:**
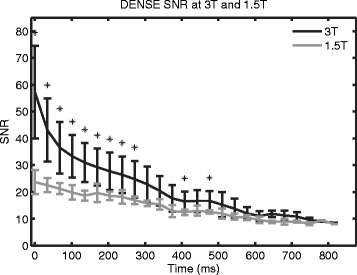
**Spiral cine DENSE SNR at 3T and 1.5T.** Average SNR curves from all subjects were calculated at 3T and 1.5T. Spiral cine DENSE imaging was performed with a constant 20° flip angle. The SNR at 3T was higher than at 1.5T through 750 ms. Statistical significance (p < 0.05) is indicated by asterisks. The last significant difference occurred at 476 ms.

The SNR at 3T remained higher than the SNR at 1.5T for 750 ms (Figure 8). This difference was significant for periods up to 476 ms by the Wilcoxon rank sum test (p < 0.05). Using all of the cardiac phases, the SNR at 3T was greater than the SNR at 1.5T by a factor of 1.4 ± 0.3.

**Figure 9:**
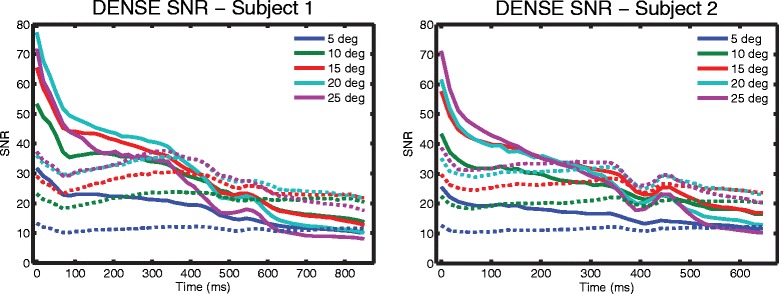
**DENSE SNR of different flip angle strategies in two subjects at 3T.** Spiral cine DENSE of a mid-ventricular slice was performed in two subjects with different flip angle strategies. The ramped flip angle strategies (dashed lines) ramp up to the indicated degree in the legend. Constant flip angle strategies (solid lines) provided high SNR in early systole at the expense of diastole. Ramped flip angle strategies (particularly 15°, 20°, and 25°) provided SNRs above 20 for most cardiac phases.

Rather than using a constant 20° flip angle, the SNR gain at 3T may be better leveraged with a ramped flip angle strategy. To investigate this, SNR was measured in two subjects for a range of constant and ramped flip angles at 3T. In Figure 9, the solid lines represent the SNR curves of constant flip angle strategies while the dashed lines represent the SNR for the ramped flip angle strategies. In both subjects, the constant flip angle strategies provided higher SNR (except for the 5° case) in early systole. However, they had the lowest SNRs in diastole. The ramped flip angle strategies, particularly the 15°, 20°, and 25° cases, provided SNRs above 20 for most cardiac phases in both subjects.

## Discussion

Spiral cine DENSE has been validated and utilized at 1.5T for measuring cardiac displacements and deformation [3-5]. In the present study, we investigated the hypothesis that the same spiral acquisition could be used at 3T to gain SNR without compromising displacement accuracy due to increased field inhomogeneities or off resonance effects that are likely present at the higher field strength. We developed a displacement validation technique that used DENSE and tagged images to measure the error in physiologic displacements from human volunteers. Our primary findings included: 1) the displacement error in spiral cine DENSE at 3T was less than the DENSE pixel spacing and not different from the displacement error at 1.5T; 2) the inter-observer variability of peak strains and torsion from spiral cine DENSE at 3T was acceptable and comparable to the inter-observer variability at 1.5T; 3) The agreement between spiral cine DENSE at 3T and HARP-based analysis of tagged images was acceptable for circumferential and longitudinal strains and comparable to the agreement at 1.5T for all strains and torsion; and 4) the SNR of spiral cine DENSE was higher at 3T and may be best leveraged with ramped flip angle strategies that maintain SNR throughout the cardiac cycle.

### Displacement validation

Displacement validation at 3T was performed *in vivo* in order to investigate the accuracy of spiral cine DENSE in the setting of physiologic cardiac displacements. Validating the displacements *in vivo* had the added benefit of being affected by the field inhomogeneities caused by the human form, which would not be present when imaging a displacement phantom. Tagged images were acquired to define the ground truth cardiac motion and deformation. Points plotted at the intersection of tag lines near end-systole formed a deformed grid. Eulerian displacements obtained from DENSE phase images ideally contained the exact information needed to project these deformed points back into the initial perfect grid. The deviation from a perfect grid, as measured by RMSE, was a measure of the accuracy in the DENSE displacements. The present method used limited post-processing that included no smoothing of the displacements. The goal was to investigate the accuracy of raw data from DENSE without introducing confounding post-processing techniques. At 3T, the DENSE errors were less than both the tag spacing of 8 mm and the DENSE pixel spacing of 2.8 mm. Many of the errors were less than the tag pixel spacing of 1.3 mm. In addition, there were no significant differences in displacement error between spiral cine DENSE at 3T and spiral cine DENSE at 1.5T. Due to the novelty of this validation technique, comparable results for physiologic displacements were not found in the literature. However, the small magnitude of these errors and the similarity between 3T and 1.5T suggest that any field inhomogeneities or off resonance effects did not substantially affect the measured displacements at the higher field strength.

### Strain and torsion analyses

The peak strains obtained from spiral cine DENSE at 3T were comparable to the results obtained at 1.5T and to the results of other studies of cardiac mechanics in humans [3,22]. In particular, Young *et al.* performed spiral cine DENSE at 1.5T on 19 healthy volunteers and reported mid-ventricular circumferential strain and radial strain to be −18.3% and 36.6%, respectively. At 3T in the present study, those values were −18.1% and 28.4% (Table 3).

**Table 3:** **Peak strains and torsions from DENSE and HARP at 3T and 1.5T (mean ± standard deviation)**

	**3T**			**1.5T**		
	**DENSE Obs 1**	**DENSE Obs 2**	**HARP**	**DENSE Obs 1**	**DENSE Obs 2**	**HARP**
**Circumferential strain (%)**						
Base	−15.9 ± 1.8	−16.4 ± 1.6	−16.4 ± 2.6	−15.0 ± 2.3	−16.6 ± 2.7	−16.5 ± 2.0
Mid	−18.1 ± 2.1	−19.0 ± 2.3	−17.9 ± 1.9	−18.2 ± 0.8	−18.7 ± 0.8	−18.2 ± 1.6
Apex	−21.0 ± 2.4	−22.2 ± 2.7	−20.4 ± 3.2	−19.7 ± 1.9	−21.4 ± 2.5	−21.9 ± 1.4
**Radial strain (%)**						
Base	52.2 ± 12.6	61.4 ± 21.1	45.8 ± 18.5	62.4 ± 24.0	60.9 ± 26.0	47.9 ± 18.0
Mid	28.4 ± 7.1	29.7 ± 8.6	34.0 ± 26.4	41.6 ± 10.1	33.7 ± 8.1	30.7 ± 10.5
Apex	29.2 ± 7.7	31.0 ± 11.4	45.9 ± 13.4	34.6 ± 14.9	30.6 ± 13.4	49.5 ± 19.5
**Longitudinal strain (%)**						
Four-chamber	−14.7 ± 1.4	−14.3 ± 1.7	−13.0 ± 1.2	−12.3 ± 1.8	−13.1 ± 1.3	−14.3 ± 2.7
**Torsion (deg/mm)**						
Global	0.38 ± 0.07	0.39 ± 0.07	0.24 ± 0.07	0.31 ± 0.06	0.32 ± 0.06	0.19 ± 0.05

The inter-observer variability of strains and torsion from spiral cine DENSE at 3T were good for longitudinal strain, circumferential strain, and torsion while acceptable for radial strain. The variability at 3T was comparable to that at 1.5T and to the variability reported in the literature [3]. Young *et al.* reported inter-observer 95% limits for circumferential strain and radial strain of ±2.3% and ±6.9%, respectively. At 3T in the present study, we found those limits to be ±1.4% and ±14.7%, respectively.

The agreement of strains and torsion between DENSE and HARP was similar between 3T and 1.5T in the present study. Larger biases and variability were seen for torsion and radial strain compared to longitudinal strain and circumferential strain. Higher variability is expected for radial strain as this parameter is known to be less robust to quantify than the other parameters [22]. The 95% limits of this agreement for all measurements were larger than the inter-observer variability of DENSE but were comparable to previous results [3]. Young *et al.* compared circumferential and radial strains between DENSE and tags by using a generalized analysis framework rather than HARP. They reported 95% limits for circumferential strain and radial strain of ±3.9% and ±14%. The present study found similar limits for circumferential strain (±4.8%) but higher limits for radial strain (±40.0%). The difference in limits between the two studies may be due to differences between HARP and the generalized analysis framework.

The similarities between 3T and 1.5T in strain values, inter-observer variability, and agreement with HARP suggest that any field inhomogeneities or off resonance effects did not lead to additional errors in quantification of cardiac displacements and mechanics at 3T.

### DENSE signal to noise ratio and flip angle analyses

This study used constant 20° flip angles to compare spiral cine DENSE between 3T and 1.5T because that flip angle strategy was prevalent in the literature for 1.5T [3,10,11]. With these constant flip angles, the SNR at 3T was 40% higher than the SNR at 1.5T. At 3T, the ramped flip angles strategies of 15°, 20°, and 25° provided SNRs above 20 for most cardiac phases. At 1.5T and with the typical constant 20° flip angle strategy, only the first few cardiac phases had SNRs above 20 while the SNR at end-systole was near 13 and the SNR in diastole was near 9. Future studies with spiral cine DENSE at 3T may use a ramped flip angle strategy to better evaluate cardiac mechanics later in the cardiac cycle with about 100% improvement in diastolic SNR compared to the constant flip angle strategy at 1.5T.

### Limitations

The low number and healthy nature of the subjects may limit the applicability of these results to different patient populations. However, the cardiac deformation that is present in healthy subjects is likely larger than that found in most patients. The deformations present in this study were a reasonable test of the accuracy of spiral cine DENSE at 3T.

The tagged images were used to define the true motion of the tag intersection points, rather than using a deformable phantom with an externally verified displacement field. There was likely some variability in the manual identification of the tag intersection points. The use of tagged images was necessary because of the difficulty in producing phantoms with known, physiologic deformations. A phantom was also not likely to recreate the field inhomogeneities that are present due to the human form.

A previous study in mice at 7T describes a method for determining the location of the initial grid of tag intersection points by relying on the tag spacing in stationary tissue [23]. This method was inadequate for our study, particularly at 3T, where much of the stationary tissue was located outside of the adjust volume where proper shimming was not performed and tag lines were significantly warped.

While DENSE has been extended to measure displacements in three dimensions, only 2D (in-plane) displacements were investigated in this study [5,16,24]. Many of the applications of DENSE within patient populations have utilized only in-plane displacements [25,26]. Furthermore, displacement errors due to field inhomogeneities and off resonance effects should be adequately assessed by investigating the in-plane displacements. While cardiac motion does contain a substantial through-plane component, this component was not required for the method of displacement validation. The in-plane displacements were sufficient for projecting deformed tag intersection points into the original 2D grid regardless of the longitudinal motion that occurred.

### Future directions

The primary findings of the present study indicate that the current form of spiral cine DENSE can be implemented at 3T without modifications to compensate for the higher field strength. Future studies can take immediate advantage of the additional SNR at 3T, which may be applied during diastole if a ramped flip angle strategy is used. Alternatively, the additional SNR may be allocated to increased spatial resolution.

## Conclusions

Cine DENSE has inherently low SNR due to the stimulated echo acquisition that has been partially offset with a spiral acquisition [1,2,5,6]. This spiral acquisition has been validated and used extensively at 1.5T, where field inhomogeneities and off resonance effects are smaller than at 3T. We demonstrated that the same spiral cine DENSE acquisition can be used at both 1.5T and 3T with equivalent accuracy. Furthermore, the inter-observer variability and agreement with HARP was comparable at both field strengths. Future studies with spiral cine DENSE may take advantage of the additional SNR at 3T.

## Abbreviations

CMR: Cardiovascular magnetic resonance
CoV: Coefficient of variation
CSPAMM: Complementary spatial modulation of magnetization
DENSE: Displacement encoding with stimulated echoes
ECG: Electrocardiogram
EPI: Echo planar imaging
HARP: Harmonic phase
LV: Left ventricle (ventricular)
MRI: Magnetic resonance imaging
RMSE: Root-mean-squared error
SNR: Signal to noise ratio
